# Identification and functional evaluation of the reductases and dehydrogenases from *Saccharomyces cerevisiae* involved in vanillin resistance

**DOI:** 10.1186/s12896-016-0264-y

**Published:** 2016-04-01

**Authors:** Xinning Wang, Zhenzhen Liang, Jin Hou, Xiaoming Bao, Yu Shen

**Affiliations:** State Key Laboratory of Microbial Technology, Shandong University, Shan Da Nan Road 27, Jinan, 250100 China

**Keywords:** Budding yeast, Vanillin tolerance, *ADH6*, *YNL134C*, *YJR096W*

## Abstract

**Background:**

Vanillin, a type of phenolic released during the pre-treatment of lignocellulosic materials, is toxic to microorganisms and therefore its presence inhibits the fermentation. The vanillin can be reduced to vanillyl alcohol, which is much less toxic, by the ethanol producer *Saccharomyces cerevisiae*. The reducing capacity of *S. cerevisiae* and its vanillin resistance are strongly correlated. However, the specific enzymes and their contribution to the vanillin reduction are not extensively studied. In our previous work, an evolved vanillin-resistant strain showed an increased vanillin reduction capacity compared with its parent strain. The transcriptome analysis suggested the reductases and dehydrogenases of this vanillin resistant strain were up-regulated. Using this as a starting point, 11 significantly regulated reductases and dehydrogenases were selected in the present work for further study. The roles of these reductases and dehydrogenases in the vanillin tolerance and detoxification abilities of *S. cerevisiae* are described.

**Results:**

Among the candidate genes, the overexpression of the alcohol dehydrogenase gene *ADH6*, acetaldehyde dehydrogenase gene *ALD6*, glucose-6-phosphate 1-dehydrogenase gene *ZWF1*, NADH-dependent aldehyde reductase gene *YNL134C*, and aldo-keto reductase gene *YJR096W* increased 177, 25, 6, 15, and 18 % of the strain μ_max_ in the medium containing 1 g L^−1^ vanillin. The in vitro detected vanillin reductase activities of strain overexpressing *ADH6*, *YNL134C* and *YJR096W* were notably higher than control. The vanillin specific reduction rate increased by 8 times in *ADH6* overexpressed strain but not in *YNL134C* and *YJR096W* overexpressed strain. This suggested that the enzymes encoded by *YNL134C* and *YJR096W* might prefer other substrate and/or could not show their effects on vanillin on the high background of Adh6p in vivo. Overexpressing *ALD6* and *ZWF1* mainly increased the [NADPH]/[NADP^+^] and [GSH]/[GSSG] ratios but not the vanillin reductase activities. Their contribution to strain growth and vanillin reduction were balancing the redox state of strain when vanillin was presented.

**Conclusions:**

Beside the reported Adh6p, the enzymes encoded by *YNL134C* and *YJR096W* were proved to have vanillin reduction activity in present study. While *ALD6* and *ZWF1* did not directly reduce vanillin to vanillyl alcohol, their contribution to vanillin resistance primarily depended on the enhancement of the reducing equivalent supply.

**Electronic supplementary material:**

The online version of this article (doi:10.1186/s12896-016-0264-y) contains supplementary material, which is available to authorized users.

## Background

Lignocellulosic materials are a readily available and renewable resource for biofuel and chemical production instead of starch, sucrose, or other resources that are better suited as food. Pretreating the lignocellulosic materials, generally with diluted acid at a high temperature, is necessary to overcome the recalcitrant structure of lignocellulose and separate the cross-linked polysaccharides. However, with the release of sugars, toxic compounds, such as organic acids (acetic acid and formic acid), furans [furfural and 5-hydroxymethyl furfural (HMF)], and phenolics are produced during the pretreatment process [1, 2]. These toxic compounds inhibit the growth and fermentation efficiency of the microorganisms.

*Saccharomyces cerevisiae* is recognized as a traditionally competitive cell factory for biorefining because of its superior tolerance to ethanol and low pH and its ease of genetic manipulation [3–5]. The resistance of *S. cerevisiae* to organic acids and furans was extensively investigated. Acetic acid enters yeast cells and causes a decrease of pH in the cytoplasm, inhibition of metabolism, and disruption of the proton gradient of the plasma membrane [6]. Inhibiting the plasma membrane channel Fps1p used for uptake of acetate and increasing the expression of major facilitator superfamily and ATP-binding cassette transporters, which are responsible for acetate excretion, increases the resistance of *S. cerevisiae* to acetic acid [6, 7]. Furans cause reactive oxygen species (ROS) accumulation in cells and decrease energy production by inhibiting glycolysis, which prolongs the lag phase [8–11]. Increasing the expression of Adh6p, Adh7p, Ald4p, Gre3p, Adh1p, Ari1p, and Gre2p, which have furfural or HMF reductase activity, or Zwf1p, Gnd1p, Gnd2p, Tdh1p, and Ald6p, which increase the NADPH supply, enhanced the rate of furfural and HMF detoxification in *S. cerevisiae* [12]*.* By comparison, only limited knowledge of *S. cerevisiae* tolerance to phenolics is reported.

Phenolic compounds, which are generated from the segmental degradation of lignin exhibit strong detrimental effects, even at low concentrations, on the fermentation of *S. cerevisiae* [2, 13]*.* This type of compound generally suppressed growth and ethanol production rate but had little effect on the ethanol yields (Y_EtOH_). Three kinds of phenolics that contain para-hydroxyphenyl, guaiacyl, and syringyl, respectively, exist in lignocellulose hydrolysate. In general, the most toxic to least toxic of these phenolics in order is para-hydroxyphenyl > guaiacyl > syringyl. Adding a methoxy group to the aromatic ring can reduce the toxicity of phenolics by decreasing their hydrophobicity [2]. Low-molecular-mass phenolic compounds are more potent inhibitors towards *S. cerevisiae* than high-molecular-weight phenolics [9]. Vanillin is a simple guaiacyl phenol with high toxicity. At low concentrations, it is a more potent repressor of fermentation than other phenolic by-products derived from lignin [2]. Moreover, the de novo synthesis of vanillin, a common additive of foods and cosmetics, has been recently achieved in yeast cells [14]. Enhancing the strain resistance to vanillin is an important issue to achieve efficient vanillin production [14]. It was reported that vanillin triggers the accumulation of ROS in cells, fragments the mitochondria [14, 15], and represses the translation process by blocking ribosomes assembly, which cause the accumulation of processing bodies and stress granules [16]. Increasing the ergosterol level of *S. cerevisiae* enhanced the fluidity and stability of the membrane, improving the strain growth in the presence of vanillin [17]. Converting the vanillin to vanillyl alcohol, which is less toxic than vanillin, by reductases is another important and efficient way for vanillin detoxification in yeast [5, 12]. Thus, it is of interest to identify the proteins that function in vanillin reduction while minimizing any reduction in ethanol yield or increasing the production of by-products.

In our previous study, the oxidoreductase activity of the vanillin-tolerant *S. cerevisiae* strain EMV-8 was found to be significantly higher than its parent strain NAN-27 [5]. In the present work, the significantly up-regulated reductases and dehydrogenases in EMV-8 were identified and their roles in the detoxification of vanillin were characterized. The effect of overexpressing these genes on the ethanol fermentation was also studied.

## Results

### Up-regulated reductases in vanillin-tolerant *S. cerevisiae*

The vanillin-tolerant strain EMV-8 obtained by adaptive evolution exhibited a high vanillin reduction rate and antioxidant capacity. The transcriptional analysis of EMV-8 revealed an up-regulated oxidoreductase activity (GO: 0016491), which might convert the vanillin directly or supply the reduced coenzyme. To identify the roles of various dehydrogenases in vanillin tolerance, the up-regulated reductases and dehydrogenases in EMV-8 were selected as studying candidates (Table 1), as well as the gene *ADH6*, whose vanillin reductase activity has been previously identified [18].

**Table 1:** The up-regulated reductases in vanillin-tolerant *S. cerevisiae*

Genes	Log2(FC)^a^	Function of genes
*ARA1*	2.455	NADP+ dependent arabinose dehydrogenase
*ARA2*	2.715	NAD-dependent arabinose dehydrogenase
*BDH1*	2.394	NAD-dependent (R,R)-butanediol dehydrogenase catalyzes oxidation of (R,R)-2,3-butanediol to (3R)-acetoin
*BDH2*	4.594	Putative medium-chain alcohol dehydrogenase with similarity to BDH1
*YNL134C*	2.298	NADH-dependent aldehyde reductase involved in detoxification of furfural
*YJR096W*	2.264	Xylose and arabinose reductase
*ALD6*	2.875	Cytosolic aldehyde dehydrogenase which prefers NADP+, conversion of acetaldehyde to acetate
*ZWF1*	1.773	Glucose-6-phosphate dehydrogenase, catalyzes the first step of the pentose phosphate pathway
*IDP3*	2.510	Peroxisomal NADP-dependent isocitrate dehydrogenase, catalyzes oxidation of isocitrate to alpha-ketoglutarate with the formation of NADP(H+)
*MDH3*	1.198	Peroxisomal malate dehydrogenase, catalyzes interconversion of malate and oxaloacetate
ADH6^b^	−0.934	NADPH-dependent medium chain alcohol dehydrogenase with broad substrate specificity

^a^FC: Fold change, False discovery rate (FDR) of listed genes is <0.001

^b^Reported as a sole vanillin reductase in *S. cerevisiae* [17]

### Adh6p is an efficient but not sole protein with vanillin reduction activity in *S. cerevisiae*

It was reported that purified Adh6p has vanillin reduction activity *in vitro* [18]. Furthermore, vanillin breakdown was not observed in an *adh6Δ* strain [16]. Therefore, it was suggested that the vanillin conversion in *S. cerevisiae* was only dependent on Adh6p [16]*.* However, the transcriptional level of *ADH6* in the vanillin-tolerant strain EMV-8 was not higher than its parent strain NAN-27 (Table 1). Therefore, Adh6p did not contribute to increasing the vanillin reduction rate in EMV-8.

**Fig. 1:**
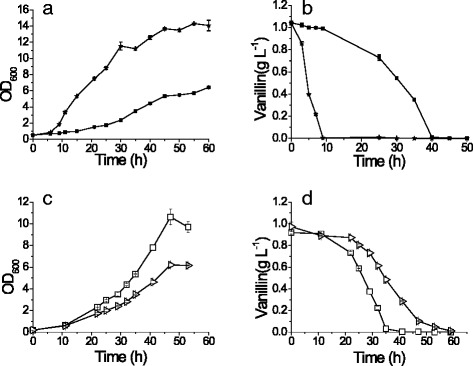
Overexpression of *ADH6* accelerated the growth in the presence of vanillin. The growth curve (**a**) and vanillin reduction (**b**) of strain BSPJ3 (containing empty vector, control; ■) and the strain overexpressing *ADH6* (★) in the SC-URA medium with 1 g L^−1^ vanillin. Comparison of growth (**c**) and vanillin reduction (**d**) of *ADH6* knock-out strain (▷) and CEN.PK102-3A (control; □) in the SD medium with 1 g L^−1^ vanillin. All the data are the mean value ± standard deviation of independent duplicate tests

The role of Adh6p in vanillin tolerance was further studied in the lab strain CEN.PK102-3A. Overexpressing *ADH6* increased the NADPH-dependent vanillin reduction activity by 97 % and remarkably enhanced the strain growth in the medium containing vanillin (Fig. 1a, Table 2). The strain overexpressing *ADH6* reduced 1 g L^−1^ vanillin to vanillyl alcohol in 9 h compared with 40 h for the control strain (Fig. 1b). Deletion of *ADH6* decreased the growth of the strain in vanillin (Fig. 1c) where the reduction of vanillin was delayed but not eliminated. The *adh6Δ* mutant reduced 1 g L^−1^ vanillin completely in 54 h (Fig. 1d). Our results revealed that Adh6p is an important and efficient vanillin reductase, but there are other reductases capable of acting on vanillin in *S. cerevisiae*.

**Table 2:** Maximum specific growth rate and ethanol yield of recombinant strains^a^

Strains^b^	SD medium		SD with 1 g L^−1^ vanillin		
	μ_max_ (h^−1^)	Ethanol yield (g g^−1^)	μ_max_ (h^−1^)^c^	Ethanol yield (g g^−1^)	Vanillin specific reduction rate (g g^−1^ h^−1^)
BSJP3	0.435 ± 0.009	0.400 ± 0.009	0.061 ± 0.001	0.340 ± 0.020	0.065 ± 0.002
*ADH6*	0.388 ± 0.032	0.390 ± 0.002	0.169 ± 0.001**	0.425 ± 0.003	0.584 ± 0.003**
*YNL134C*	0.420 ± 0.004	0.382 ± 0.004	0.070 ± 0.001**	0.380 ± 0.014	0.066 ± 0.000
*YJR096W*	0.424 ± 0.007	0.388 ± 0.007	0.072 ± 0.001**	0.379 ± 0.009	0.064 ± 0.001
*ALD6*	0.406 ± 0.013	0.363 ± 0.013	0.076 ± 0.002**	0.330 ± 0.001	0.052 ± 0.001*
*ZWF1*	0.405 ± 0.007	0.398 ± 0.007	0.065 ± 0.003*	0.347 ± 0.013	0.082 ± 0.003*

^a^The data were show as mean ± standard deviation

^b^The gene names here represent the strains overexpressing the gene. BSJP3 contains the empty vector and was used as the reference strain

^c^ **: *p* < 0.01; *: *p* < 0.1

### Overexpressing *YNL134C, YJR096W, ALD6,* and *ZWF1* enhanced strain growth in vanillin

The up-regulated genes in EMV-8 (Table 1) were overexpressed in strain CEN.PK102-3A. The empty vector transformed into CEN.PK102-3A resulted in BSJP3, which was used as the control.

**Fig. 2:**
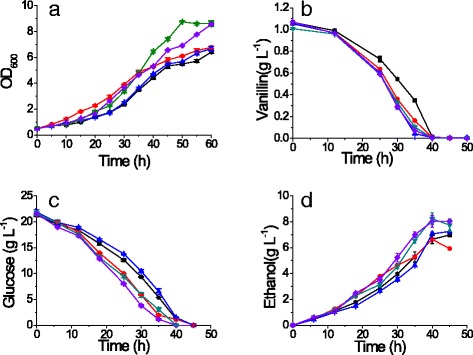
The growth and fermentation characteristics of strains. The cell growth (**a**), vanillin reduction (**b**), glucose consumption (**c**), and ethanol production (**d**) curves are shown. The fermentation was conducted in SC-URA with 1 g L^−1^ of vanillin. All the data are the mean value ± standard deviation of independent duplicate tests. Symbols: BSPJ3 (control, *Black square*); strains overexpressing *ALD6* (*Red circle*), *ZWF1* (*Blue triangle*), *YNL134C* (*Green triangle*), and *YJR09W* (*Violet diamond*)

**Fig. 3:**
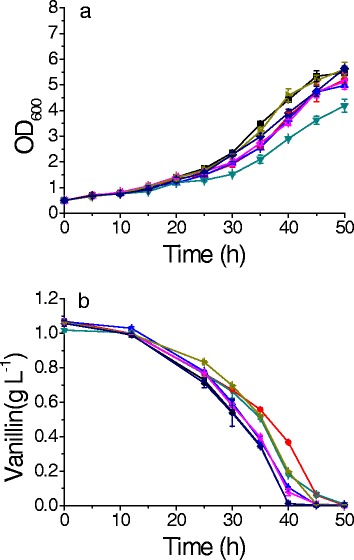
The growth curve (**a**) and vanillin reduction (**b**) of strains overexpressing *ARA1* (*Red circle*); *ARA2* (*Blue triangle*); *BDH1* (*Blue green triangle*); *BDH2* (*Pink triangle*); *MDH3* (*Yellow green triangle*); *IDP3* (*Violet diamond*) and the control BSPJ3 (■*Black square*) in the medium of SC-URA with additional 1 g L^−1^ vanillin. All the data were mean value ± standard deviation of independent two experiments

Overexpressing *YNL134C*, *YJR096W*, and *ALD6* slightly decreased the growth and ethanol yield of the resulting strains in vanillin-free medium (Table 2). However, when vanillin was present, overexpressing *YNL134C*, *YJR096W*, and *ALD6* accelerated glucose consumption and ethanol production (Fig. 2). The maximum specific growth rates of the strains overexpressing *YNL134C*, *YJR096W*, and *ALD6* were respectively 15, 18, and 25 % faster than that of BSJP3, which only contains empty vector, in the presence of 1 g L^−1^ vanillin. The ethanol yields of the strains overexpressing *YJR096W* and *YNL134C* both were 11 % higher than that of BSJP3 (Table 2). The vanillin specific reduction rates of the strains overexpression *ADH6, ALD6, ZWF1, YNL134C* and *YJR096W* were 0.584, 0.052, 0.082, 0.066, and 0.064 g g^−1^ h^−1^, respectively. Compared to the 0.065 g g^−1^ h^−1^ of control, only *ADH6* and *ZWF1* significantly increased the specific vanillin reduction rate. Other genes may accelerate growth more than vanillin reduction. Overexpressing the genes *ARA1, ARA2, BDH1, BDH2, MDH3,* and *IDP3*, which were up-regulated in the vanillin-tolerant strain EMV8, did not enhance strain growth in the presence of vanillin (Fig. 3).

**Fig. 4:**
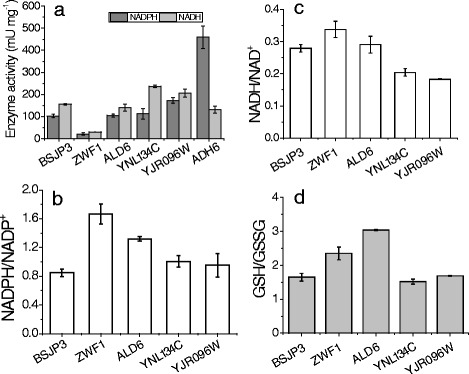
The crude enzyme activities (**a**); the ratio of NADPH to NADP^+^ (**b**); the ratio of NADH to NAD^+^ (**c**); and ratio of cellular GSH to GSSG (**d**) in recombinant strains. The fermentation was conducted in SC-URA medium. All data are the mean value ± standard deviation of independent duplicate tests

The vanillin reductase activity, the ratio of NAD(P)H to NAD(P)^+^, and the ratio of reduced glutathione (GSH) to oxidized glutathione (GSSG) of the strains were determined to distinguish whether the growth improvement was due to the enhanced vanillin reductase activity or the increased reduced-coenzyme and GSH supply. The enzymes encoded by *ADH6*, *YNL134C* and *YJR096W* prefer different coenzymes and exhibited notable vanillin reduction activity in vitro (Fig. 4a). The reductase activity encoded by *ADH6* mainly depended on NADPH. Overexpressing *ADH6* increased the vanillin reductase activity of strain by 3.5 times. The reductase activity encoded by *YNL134C* mainly depended on NADH. The strain overexpressing *YNL134C* exhibited 153 % of the NADH-dependent vanillin reduction activity of the control strain. The enzyme encoded by *YJR096W* had both NADH- and NADPH-dependent vanillin reduction activities. Overexpressing *YJR096W* increased strain NADH- and NADPH-dependent vanillin reduction activity by 45 and 69 %, respectively, while the Ald6p and Zwf1p did not contribute to the vanillin reduction activity of the test strains (Fig. 4a). The ratio of NADPH to NADP^+^ from the strains overexpressing *ZWF1*, *ALD6, YNL134C*, and *YJR096W* were 1.96, 1.55, 1.17, and 1.12 times the control, respectively (Fig. 4b). The ratio of NADH to NAD^+^ from the strains overexpressing *ZWF1* also increased 21 % compared to the control strain (Fig. 4c). Furthermore, the ratio of GSH to GSSG from the strains overexpressing *ZWF1*, *ALD6*, *YNL134C*, and *YJR096W* were 1.4, 1.8, 0.9, and 1.0 times the control strain, respectively (Fig. 4d). This suggested that overexpressing *ZWF1* and *ALD6* enhanced the antioxidant capacity of the yeast strain.

## Discussion

The vanillin tolerance of *S. cerevisiae* can be primarily attributed to its antioxidative and vanillin detoxification capacity [15]. The vanillin detoxification in yeast depends on the reduction of vanillin [12]. In the present study, we confirmed that overexpressing *ADH6*, *YNL134C*, *YJR096W*, *ALD6*, and *ZWF1* enhanced strain growth and accelerated vanillin reduction in the presence of vanillin. The enzymes encoded by *ADH6*, *YNL134C*, and *YJR096W* show in vitro detected vanillin reductase activities, however, only *ADH6* increased the vanillin specific reduction rate. The enzymes encoding by *YNL134C* and *YJR096W* may have more prefer substrates in vivo during the fermentation. For example, the acetaldehyde was one of favorite substrates of enzyme encoding by *YNL134C* [19]. Furthermore, effects of *YNL134C* and *YJR096W* could be concealed on the high background activity of Adh6p. Therefore, overexpressing *YNL134C* and *YJR096W* did not increase the vanillin specific reduction rate. However, their whole accumulative contribution on vanillin detoxification should not be underestimated. *YJR096W* were found to response to DNA damage [20] and *YNL134C* encodes a non-zinc-containing MDR (medium-chain dehydrogenases/reductases), which type of proteins behaved protective functions [19, 21]. These characteristics may related to the enhancement of strain growth in the medium containing vanillin, but the mechanism is still not clear. While overexpression of *ALD6* and *ZWF1* enhanced the NADPH supply, and increased the ratio of cellular GSH to GSSG, which helped to improve the antioxidant activity of the strain.

Certain aldehydes, such as furfural and HMF that derived from sugars and various phenolics that generated from the breakdown of lignin, are the main toxic compounds in lignocellulose hydrolysates. Their detoxification is directly related to their reduction. Therefore, the organism may benefit more from a multifunctional reductase than an enzyme specific to one or two aldehydes. The enzymes encoded by *ADH6*, *YNL134C*, and *YJR096W* all accept a wide range of substrates. The purified Adh6p possessed a specific NADPH-dependent reductase activity and accepted a broad range of substrates, including linear and branched-chain primary alcohols and aldehydes, substituted cinnamyl alcohols and aldehydes, as well as substituted benzaldehydes and their corresponding alcohols [18]. Its high reduction activities towards furfural and HMF were also demonstrated [12, 22]. The enzyme encoded by *YNL134C* was previously described as a NADH-dependent aldehyde reductase with activity towards formaldehyde, acetaldehyde, and furfural [19]. The enzyme encoded by *YJR096W* is known as an arabinose reductase [23]. It also has high catalytic efficiencies towards aromatic aldehydes such as benzaldehydes and phenylglyoxal [24]. Therefore, these genes may be of use to increase the strain tolerance to lignocellulosic materials. The reduced coenzyme is another limiting factor involved in aldehyde reduction. Zwf1p catalyzes the first step (also the rate-limiting step) of the pentose phosphate pathway, which is the primary route for generating NADPH in yeast [25, 26]. Ald6p provides NADPH by converting acetaldehyde to acetate [26]. Overexpressing *ALD6* and *ZWF1* increases the intracellular NADPH radio, therefore enhancing the reduction of furfural, HMF [27], and vanillin in our work. NADPH is also an electron acceptor of GSH regeneration [28]. The GSH is the main component of the antioxidant system in living cells, scavenging ROS by oxidizing to GSSG. The increased ratio of GSH to GSSG may help to protect the cells from intracellular ROS accumulation induced by vanillin [15]. Therefore, overexpressing *ALD6* and *ZWF1* may be a general strategy to increase the strain tolerance to lignocellulosic materials. However, because the generation of NADPH is coupled with carbon flow to the pentose phosphate pathway and pathways generate byproducts, further effort is required to fine tune the expression of these genes to balance the product yield and strain tolerance.

## Conclusions

The enzymes encoded by *ADH6*, *YNL134C*, and *YJR096W* have vanillin reductase activity, while overexpression of *ALD6* and *ZWF1* had positive effects on vanillin resistance and detoxification in *S. cerevisiae*, which was attributed to the enhancement of the NADPH supply. Overexpressing these genes enhanced the growth and metabolism of *S. cerevisiae* in environment containing the inhibitor vanillin. Additionally, since the benefits of those genes to strain tolerance towards other toxic compounds found in lignocellulosic hydrolysates, such as furfural, were suggested, overexpression of select genes may represent a general strategy for increasing the tolerance of *S. cerevisiae* to lignocellulosic materials and warrants further investigation.

## Methods

### Construction of plasmids and yeast strains

The genes expressed in this work were amplified from the genomic DNA of the lab strain CEN.PK102-3A (*MATa*; *ura3-52*, *His3Δ1*, *leu2-3,112*) [29]. The 2 μ plasmid pJFE3 [30] was transformed into CEN.PK102-3A to yield BSPJ3, which was used as the control. The genes were inserted into plasmid pJFE3 under the control of the *TEF1* promoter and *PGK1* terminator. The resulting recombinant plasmids were transformed into strain CEN.PK102-3A to yield the respective gene overexpression strains. The gene deletion was performed through homologous recombination. The primers (Additional file 1: Table S1) used to amplify the destruction cassette from plasmid pUG6 contain a sequence that is homologous with the deleted gene and the KanMX expression cassette. The destruction cassette was transformed into the lab strain CEN.PK102-3A and the mutants were screened in YPD medium (10 g L^−1^ yeast extract, 20 g L^−1^ tryptone, 20 g L^−1^ glucose, pH 5.0) containing 400 mg L^−1^ G418.

### Fermentation

SD or SC-URA medium, which containing 20 g L^−1^ glucose, 1.7 g L^−1^ yeast nitrogen base (YNB, Sangon, China), and 5 g L^−1^ ammonium sulfate (Sangon, China) replenished with CSM or CSM-URA (MP Biomedicals, Solon, OH, USA), and 1 g L^−1^ vanillin was used for batch fermentation. A single colony was inoculated into 3 mL SD or SC-URA, cultured 24 h at 30 °C, transferred into 10 mL fresh medium with OD_600_ at 0.2 and cultured 12 h. Then, the cells were inoculated into 100-mL flasks containing 40 mL of fermentation medium with an initial OD_600_ of 0.5. The fermentation was performed at 30 °C and 200 rpm.

### Analyses of extracellular metabolite

The concentrations of glucose and ethanol were tested using a HPLC prominence LC-20A (Shimadzu, Japan) equipped with an Aminex HPX-87H ion exchange column (Bio-Rad, USA) and refractive index detector RID-10A (Shimadzu, Japan); 5 mmol L^−1^ H_2_SO_4_ was used as a mobile phase with flow rate of 0.6 mL min^−1^ at 45 °C [4]. Vanillin and vanillyl were also determined by HPLC using a BioSil-C18 column (Bio-Rad, USA). The peaks were detected at room temperature using ultraviolet detection (SPD-M20A) at 210 nm with a mobile phase containing 40 % aqueous methanol supplied at a flow rate of 0.6 mL min^−1^ [13].

### Calculation of physiological parameters

The biomass concentrates were estimated according to the measured *OD*_*600*_-dry weight correlation. One unit of *OD*_*600*_ equals 0.23 g L^−1^ biomass. The maximum growth rates are the linear regression coefficients of the *ln OD*_*600*_ versus time during the exponential growth phase. Specific consumption rates of vanillin were calculated using the following equation:

$$ r=\frac{A_n-{A}_m}{\frac{1}{2}{\displaystyle {\sum}_{i=m+1}^n}\left({B}_i+{B}_{i-1}\right)\times \left({t}_i-{t}_{i-1}\right)} $$

Where *r* is the specific consumption rate during the phase from sampling point *m* to sampling point *n*; *A*, *B*, and *t* are the metabolite concentration, biomass concentration, and time, respectively, at sampling points *n*, *i*, and *m*, as previously described [31]. The ethanol yields are the ethanol concentrate versus the consumed sugar.

### Enzyme activity assay

The cells were cultured in SC-URA medium. When the OD reached 4.0, the cells were harvested and re-suspended in 33 mM Na_3_PO_4_ buffer (pH = 7.0) with 1 mM PMSF, which was used to inhibit any protease activity. Then, the cells were broken by φ 0.5-mm glass beads using a Fast Prep cell homogenizer (ThermoSavant, Germany) and centrifuged at 12,000 × g for 10 min. The supernatant was collected as a cell-free extract for the enzyme activity assay.

The dehydrogenase activity towards vanillin was determined according to the method of Larroy [18]. Enzyme activities towards vanillin were assayed in 0.6-mL reaction mixtures containing 33 mM sodium phosphate buffer (pH 7.0), 0.5 mM NADPH, and 1 mM vanillin (using 0.2-cm path length cuvettes). The molar absorption coefficient (ε_365_) was 7.71 mM^−1^ cm^−1^ for vanillin plus NADPH. One unit (U) of enzyme activity is defined as the amount of enzyme that can reduce 1 μmol of NADPH plus vanillin per minute. Protein concentrations were measured using a BCA protein assay reagent kit (Beyotime, China). The specific enzyme activity (U mg^−1^ protein) was the enzyme activity per milligram of protein.

### Quantification of NAD(P)^+^ and NAD(P)H

Cells were cultured in 40 mL SC-URA medium in a 100-mL flask starting at an initial OD_600_ of 0.2. When the OD_600_ reached 4.0, 20 mL of culture were injected into 30 mL of −80 °C precooled methanol to quench the cells. Cells were collected by centrifugation at −20 °C at 12,000 × g for 5 min and washed twice with ice-cold phosphate-buffered saline (PBS). Then, the cells were resuspended in 150 μL of 0.2 M NaOH (for NAD(P)H extraction) or 150 μL of 0.2 M HCl (for NAD(P) ^+^extraction) and multi-gelated by liquid nitrogen. The extracts were neutralized by adding 150 μL of 0.1 M HCl (for NAD(P)H extraction) or 150 μL of 0.1 M NaOH (for NAD(P)^+^ extraction). The cellular debris was removed by centrifugation at 12,000 × g for 5 min. The supernatants were transferred to new tubes and stored at −80 °C until the assay.

The concentration of NADP(H) was quantified using a sensitive enzymatic cycling assay as reported previously [32, 33]. The cycling assay was carried out in a reagent mixture containing equal volumes of 1.0 M bicine buffer (pH 8.0), 30 mM glucose-6-phosphate, 40 mM EDTA (pH 8.0), 4.2 mM 3-(4,5-dimethyl-2-thiazolyl)-2,5-diphenyl-2H-tetrazolium bromide, twice the volume of 16.6 mM phenazine ethosulfate, and 3 volumes of water, previously incubated at 25 °C. Then, 0.9 mL of the reagent mixture, 50 μL neutralized extract, and 50 μL of yeast glucose-6-phosphate dehydrogenase (10 U mL^−1^, Sigma, USA) were added to start the NADP(H) assay in 1-mL cuvettes. The absorbance at 570 nm was recorded for 1 min at 25 °C. The standard curves of NADPH and NADP^+^ were conducted in the aforementioned assay buffer using a gradient concentration of NADPH or NADP^+^ instead of the neutralized extract.

For NAD(H) determination, the final concentration of 10 % (v/v) ethanol was used instead of glucose-6-phosphate in the reaction mixture, and the reaction was started by adding 50 μl of yeast ethanol dehydrogenase II (500 U mL^−1^ in Bicine buffer, Sigma, USA) instead of glucose-6-phosphate dehydrogenase.

### Quantification of GSH and GSSG

The cells were cultured in SC-URA medium and collected when the OD_600_ reached 4.0 and then were washed by PBS. The mixture of cells were added to three times the volume of deproteinization buffer M from the GSH and GSSG Assay Kit (Beyotime S0053, China) and then were alternately subjected to multi-gelation twice in liquid nitrogen and 37 °C water. After centrifugation (12,000 × g, 10 min, 4 °C), the supernatant was collected for GSH and GSSG determination. The levels of GSH and GSSG were determined using a GSH and GSSG Assay Kit (Beyotime S0053) according to the manufacturer’s protocol.

### Ethics approval and consent to participate

Not applicable.

### Consent for publication

Not applicable.

### Availability of data and material

The transcriptional dataset supporting the conclusions of this article is available in the Supplementary Materials of reference 5 (doi:10.1007/s10295-014-1515-3).

## Additional file

Additional file 1: Table S1. DNA primers used in this work. (DOCX 20 kb)

## Abbreviations

GSH/GSSG: reduced/oxidized form of glutathione
HMF: 5-hydroxymethyl furfural
NADH/NAD^+^: reduced/oxidized form of nicotinamide adenine dinucleotide
NADPH/NADP^+^: Reduced/oxidized form of nicotinamide adenine dinucleotide phosphate
ROS: reactive oxygen species
