# Supplementary material for: Identification and functional evaluation of the reductases and dehydrogenases from Saccharomyces cerevisiae involved in vanillin resistance
Source: BMC Biotechnol. 2016 Apr 1;16:31. doi: 10.1186/s12896-016-0264-y (PMC4818428; doi:10.1186/s12896-016-0264-y)
Supplement: Additional file 1: Table S1. — DNA primers used in this work. (DOCX 20 kb) [file 12896_2016_264_MOESM1_ESM.docx]

Table S1. DNA primers used in this work

| Primer | Sequence (5’-3’) | Purpose |
| --- | --- | --- |
| ZWF1up | CGTACGGATCCATGAGTGAAGGCCCCGTCAAATTC | Cloning of *ZWF1* |
| ZWF1down | CGTACGCGTCGACCTAATTATCCTTCGTATCTTCTGGC | Cloning of *ZWF1* |
| ALD6up | CGTACGGATCCATGACTAAGCTACACTTTGACAC | Cloning of *ALD6* |
| ALD6down | CGTACGCGTCGACTTACAACTTAATTCTGACAGC | Cloning of *ALD6* |
| YJR096Wup | CGTACGGATCCATGGTTCCTAAGTTTTACAAACTTTCAAACGGC | Cloning of *YJR096W* |
| YJR096Wdown | CGTACGCGTCGACTTATGGCGCGTCTGTGCATTCCCAATCG | Cloning of *YJR096W* |
| YNL134Cup | CGTACGGATCCATGTCCGCCTCGATTCCAGAAACCATG | Cloning of *YNL134C* |
| YNL134Cdown | CGTACGCGTCGACTTATTTCAAGACGGCAACCAACTTTTCGC | Cloning of *YNL134C* |
| MDH3up | CGTACGGATCCATGGTCAAAGTCGCAATTCTTG | Cloning of *MDH3* |
| MDH3down | CGTACACCTGCAGGTCATAGCTTGGAAGAGTCTAGG | Cloning of *MDH3* |
| IDP3up | CGTACGGATCCATGAGTAAAATTAAAGTTGTTCATCCCA | Cloning of *IDP3* |
| IDP3down | CGTACACCTGCAGGTTATAGTTTGCACATACCTTTCTTGTC | Cloning of *IDP3* |
| ARA1up | CGTACGGATCCATGTCTTCTTCAGTAGCCTCAA | Cloning of *ARA1* |
| ARAdown | CGTACACCTGCAGGTTAATACTTTAAATTGTCCAAGTTTGGTC | Cloning of *ARA1* |
| ARA2up | CGTACGGATCCATGGTTAATGAAAAAGTGAATCCA | Cloning of *ARA2* |
| ARA2down | CGTACACCTGCAGGTTATATCATTTCTGGATGAGGAATAC | Cloning of *ARA2* |
| BDH1up | CGTACGGATCCATGAGAGCTTTGGCATATTTCAAG | Cloning of *BDH1* |
| BDH1down | CGTACACCTGCAGGTTACTTCATTTCACCGTGATTGTTAG | Cloning of *BDH1* |
| BDH2up | CGTACGGATCCATGAGAGCCTTAGCGTATTTCGG | Cloning of *BDH2* |
| BDH2down | CGTACACCTGCAGGTCATGTGTGACGCAGTTTAGC | Cloning of *BDH2* |
| ADH6up | CGTACGGATCCATGTCTTATCCTGAGAAATTTGAAGGT | Cloning of *ADH6* and *ADH6* deletion |
| ADH6down | CGTACACCTGCAGGCTAGTCTGAAAATTCTTTGTCGTAGC | Cloning of *ADH6* and *ADH6* deletion |
| ADH6delup | ATTAAGGGTTGTCGACCTGCAGCGTACGAAGCTTCAGCTGCCAACGACTAGCGGCATCT | *ADH6* deletion |
| ADH6deldown | GAAGTTATTAGGTGATATCAGATCCACTAGTGGCCTATGCCATTTCTTACAGTGCTTTA | *ADH6* deletion |
| Kanup^a^ | CAGCTGAAGCTTCGTACGCTG | Cloning *KanMX* |
| Kandown^a^ | GCATAGGCCACTAGTGGATCTG | Cloning *KanMX* |

^a.^ All the genes were cloned from the genome of CEN.PK 102-3A. *KanMX* was cloned from pUG6.
